# Validation of Chinese Version of Polycystic Ovary Syndrome Health-Related Quality of Life Questionnaire (Chi-PCOSQ)

**DOI:** 10.1371/journal.pone.0154343

**Published:** 2016-04-28

**Authors:** Chung-Ying Lin, Huang-tz Ou, Meng-Hsing Wu, Pei-Chi Chen

**Affiliations:** 1 Department of Rehabilitation Sciences, Faculty of Health and Social Sciences, The Hong Kong Polytechnic University, Hung Hom, Hong Kong; 2 Institute of Clinical Pharmacy and Pharmaceutical Sciences, National Cheng Kung University College of Medicine, Tainan, Taiwan; 3 Department of Obstetrics and Gynecology, National Cheng Kung University College of Medicine and Hospital, Tainan, Taiwan; Weill Cornell Medical College Qatar, QATAR

## Abstract

**Objectives:**

To evaluate the responsiveness, longitudinal validity, and measurement invariance of the Chinese version of the Polycystic Ovary Syndrome Health-related Quality of Life Questionnaire (Chi-PCOSQ).

**Research Design and Method:**

This prospective study was conducted in a medical center in southern Taiwan. 102 women aged 18–45 years and diagnosed with PCOS were enrolled. Objective indicators for clinical changes of PCOS included assessing the 2-hour glucose and insulin levels before and after treatment. The responsiveness of Chi-PCOSQ and WHOQOL-BREF was analyzed using paired *t*-tests and the standard response mean. Confirmatory factor analysis was performed to assess the measurement invariance of Chi-PCOSQ.

**Results:**

With improved 2-hour glucose and insulin levels, we also found significantly increased Chi-PCOSQ total and individual domain scores (total score: *t* (49) = 5.20; *p* < 0.001, domain scores: *t* (49) = 2.72 to 3.87; *p* < 0.01), except for hair growth. Half of the domains scores (3 of 6) and the total score of Chi-PCOSQ had a medium responsiveness, but WHOQOL-BREF was not sufficiently responsive to clinical changes of PCOS. Improved PCOS-specific health-related quality of life (HRQoL), as indicated by Chi-PCOSQ scores, was significantly associated with improved 2-hour glucose and insulin levels. All indices of the data-model fit of the Chi-PCOSQ structure were satisfactory, except for the slightly high standardized root mean square residual values (0.087 to 0.088). The measurement invariance of Chi-PCOSQ was supported across time.

**Conclusion:**

Chi-PCOSQ is sufficiently sensitive in detecting clinical changes and its measurement structure is suitable for Chinese women with PCOS. It is thus a promising tool for assessing the HRQoL of ethnic Chinese women with PCOS.

## Introduction

Polycystic ovary syndrome (PCOS) is the most common endocrine disorder among women at reproductive age, affecting approximately 6–10% of such individuals [1]. Acne, hirsutism, and obesity, along with menstrual irregularity and infertility, which are typical symptoms associated with PCOS, are major sources of psychological morbidity [2–8] and can lead to a significant reduction in patients’ health-related quality of life (HRQoL) [2, 9–11]. Several international studies have assessed the HRQoL of women with PCOS [12–14]; however, research on the HRQoL of Chinese women with PCOS remains scarce. One of the main reasons is the lack of a valid instrument for assessing the health status of Chinese women affected by PCOS.

Recently, we adapted the HRQoL Questionnaire for Women with PCOS (PCOSQ) into a Chinese version with cultural adaptations. The newly developed Chinese version of PCOSQ was named as Chi-PCOSQ) [15]. Chi-PCOSQ was shown to be reliable and valid for a sample of ethnic Chinese women with PCOS [15]. The uniqueness of Chi-PCOSQ is that it incorporates three additional items (“acne”, “hair loss”, and “feel frightened of getting diabetes”) into the original PCOSQ. Acne and hair loss (androgenetic alopecia), as characterized by clinical hyperandrogenism, are common presentations of PCOS and were frequently reported in our previous study of Chinese women with PCOS [15]. Acne or hair loss problems associated with PCOS can lead to psychological disturbances (i.e., low self-esteem, depression, feelings of unattractiveness) in PCOS patients [16]. However, concerns associated with acne or hair loss are not addressed in the original PCOSQ [17]. In terms of future complications associated with PCOS, the women with PCOS are at risk for metabolic syndrome and diabetes [18]. Previous studies of Asian women with PCOS have shown that PCOS increases the risks of impaired glucose tolerance, gestational diabetes, and type 2 diabetes [19, 20]. In addition to assessing the fear of getting cancer in the original PCOSQ [17], our previous study found that Chinese women with PCOS also had a fear of getting diabetes [15]. Chi-PCOSQ was developed as a culturally specific HRQoL instrument and thus may better address the impact of PCOS on the HRQoL of Chinese women with PCOS.

It has been recognized that clinical representations of PCOS vary with ethnicity [21], and might thus lead to different impacts on HRQoL. For instance, the prevalence of hirsutism and obesity in Chinese women with PCOS appears to be lower than that in Caucasians patients [21]. Acne and hair loss are common problems reported by ethnic Chinese women with PCOS [15]. Assessing the impact of PCOS on the HRQoL of patients across ethnic groups is thus important. Chi-PCOSQ is therefore a promising instrument for assessing the burden of PCOS symptoms and the associated psychological distress on the HRQoL of Chinese women with PCOS as compared to those for patients from Western cultures.

However, information on the psychometric properties of Chi-PCOSQ is insufficient because it was developed very recently. Therefore, for Chi-PCOSQ, the responsiveness (i.e., sensitivity of Chi-PCOSQ to detect important clinical changes), longitudinal validity, and measurement invariance across time should be further addressed and explored. It has been reported that PCOSQ is responsive to the treatment effects of troglitazone (600 mg) in Emotions (standardized response mean; SRM = 0.40), Infertility (SRM = 0.57), and Menstruation (SRM = 0.69) domains [11]. Regarding longitudinal validity, weak correlations were found between the change of PCOSQ domain scores and the corresponding clinical changes (i.e., menstruation domain scores with menstrual regularity) [11]. However, the results from the original PCOSQ might not be generalized to Chi-PCOSQ, and clinicians might wonder whether Chi-PCOSQ also has good responsiveness and whether it is correlated with clinical changes. Moreover, clinicians might want to know whether the structure of Chi-PCOSQ is invariant across time even after the participants receive treatment or clinical intervention. Therefore, the measurement invariance of Chi-PCOSQ should be examined. If measurement invariance is supported, it would indicate that Chi-PCOSQ is a stable instrument for clinicians to capture the HRQoL of PCOS patients over time.

The present study thus assesses the responsiveness, longitudinal validity, and measurement invariance of Chi-PCOSQ across time for ethnic Chinese women with PCOS.

## Materials and Methods

We conducted a prospective observational study. This study involved human participants and was approved by the Institutional Review Board (IRB) of National Cheng Kung University Hospital, Tainan, Taiwan (A-ER-103-287) before commencement of the study. The IRB approved this study according to the Declaration of Helsinki

### Participants

Study participants were recruited from the Department of Obstetrics & Gynecology in National Cheng Kung University Hospital in Taiwan. All participants provided written informed consent regarding their willingness to participate in the research. They met the following inclusion criteria: (1) female, (2) aged 18–45 years, (3) ethnic Chinese, (4) competent in the Chinese language, (5) had a diagnosis of PCOS, and (6) were regularly (at least two outpatient visits before enrollment) followed at outpatient clinics. In order to identify newly diagnosed PCOS patients, we excluded patients who had a PCOS diagnosis or treatment for PCOS before the study started. We also excluded patients who: (1) were diagnosed with a similar clinical presentation, including congenital adrenal hyperplasia, androgen secreting tumors, Cushing syndrome, thyroid dysfunction, and hyperprolactinaemia, (2) were previously diagnosed with diabetes or had a fasting plasma glucose test result of > 126 mg/dL at the time of inclusion, (3) took any medication that affects insulin levels or hormonal medications, including contraceptive pills, at least two months before participating in this study, (4) were known to have suffered a major traumatic event at least 6 months prior to data collection, such as divorce, separation, or the death of someone close. All participants gave a written informed consent regarding their willingness to participate in the research. The fourth author assessed each participant, and confirmed that all participants had completed Chi-PCOSQ, World Health Organization Quality of Life-BREF (WHOQOL-BREF), and demographic questions regarding age, gender, residence, highest education level, occupation, comorbidity, and co-medications. In order to assess the responsiveness, longitudinal validity, and measurement invariance of Chi-PCOSQ, all participants were asked to fill out the questionnaires at the visit in which they were diagnosed with PCOS (baseline) and at every return visit every 1–2 months after metformin treatment (500 mg, TID) was initiated.

### Measurements

#### 2-hour post-load glucose and insulin levels

The 2-hour glucose and insulin data are recognized as objective indicators for clinical changes of PCOS and both indicators have been used to screening PCOS patients. Also, metformin efficacy has been shown to be associated with improved 2-hour glucose [22, 23] and insulin [24] levels in PCOS patients. Therefore, we used the 2-hour glucose and insulin data help us determine if the participants had any clinical changes in their PCOS (i.e., improvement because of metformin treatment). The 2-hour glucose and insulin levels were determined using a standardized 75-g oral glucose tolerance test (OGTT). The OGTT was performed to assess glucose and insulin levels at 0 and 120 minutes. The 2-hour glucose and insulin levels were measured at the baseline and every time patients returned for a visit after metformin treatment was initiated. Only data from those who had laboratory data of 2-hour glucose and insulin were used for assessing the responsiveness and longitudinal validity of Chi-PCOSQ.

#### Health-related quality of life questionnaires

Chi-PCOSQ [15] is a disease-specific HRQoL questionnaire that contains 31 questions reflecting patients’ concern or psychological disturbances associated with the symptoms of PCOS. All items were rated on a seven-point rating scale (1, maximum impairment, to 7, no impairment of HRQoL) and distributed in the following six domains: Emotions (8 items), Hair growth (5 items), Body weight (5 items), Infertility (5 items), Menstruation (5 items), and Acne & hair loss (5 items). The items of acne, hair loss, and fear of getting diabetes were specifically designed for Chinese women. Similar to the psychometric findings reported for the original PCOSQ [11, 16, 17], Chi-PCOSQ showed good test-retest reliability (all intraclass coefficients > 0.7) and acceptable internal reliability (all Conbroach’s α > 0.7) [15]. Construct validity was confirmed by significant correlation between the domains of Chi-PCOSQ and generic HRQoL measures (WHOQOL-BREF, EQ-5D) and clinical parameters (body mass index, waist-hip ratio, blood pressure) [15].

WHOQOL-BREF [25] has 26 items using a five-point Likert scale to measure HRQoL for general populations. In addition, the Taiwanese version of WHOQOL-BREF has two domestic items for cultural adaptation, for a total of 28 items. The items are distributed into two generic items and four domains: Physical health (7 items), Psychological health (6 items), Social relations (4 items), and Environment (11 items). The psychometric properties of the Taiwanese version of WHOQOL-BREF are satisfactory, including good internal consistency (Cronbach’s α = 0.70–0.91), excellent test-retest reliability (*r* = 0.76–0.80), and acceptable construct validity (comparative fit index; CFI = 0.89) [26].

### Statistical analyses

In addition to the demographics analyzed using descriptive analyses, the responsiveness of the Chi-PCOSQ domain and total scores and that of the WHOQOL-BREF domain and total scores were analyzed using paired *t*-tests and SRM. The SRM values were calculated as the mean change scores divided by the standard deviation (SD) of the change, where SRM < 0.2 is trivial, 0.2 to 0.5 is small, 0.5 to 0.8 is medium, and > 0.8 is large based on the guidelines from Middle and van Sondern [27]. Moreover, we determined the correlations between the change of the 2-hour glucose/insulin levels and that of the PCOSQ total and domain scores to examine longitudinal validity.

**Fig 1:**
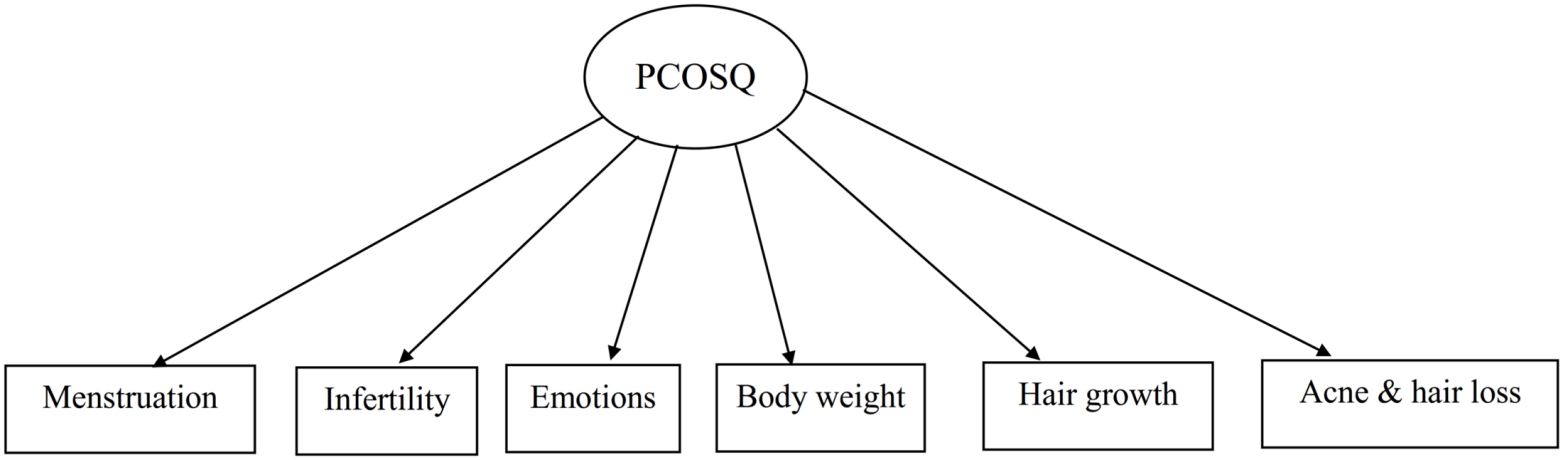
Priori structure for Chi-PCOSQ.

**Fig 2:**
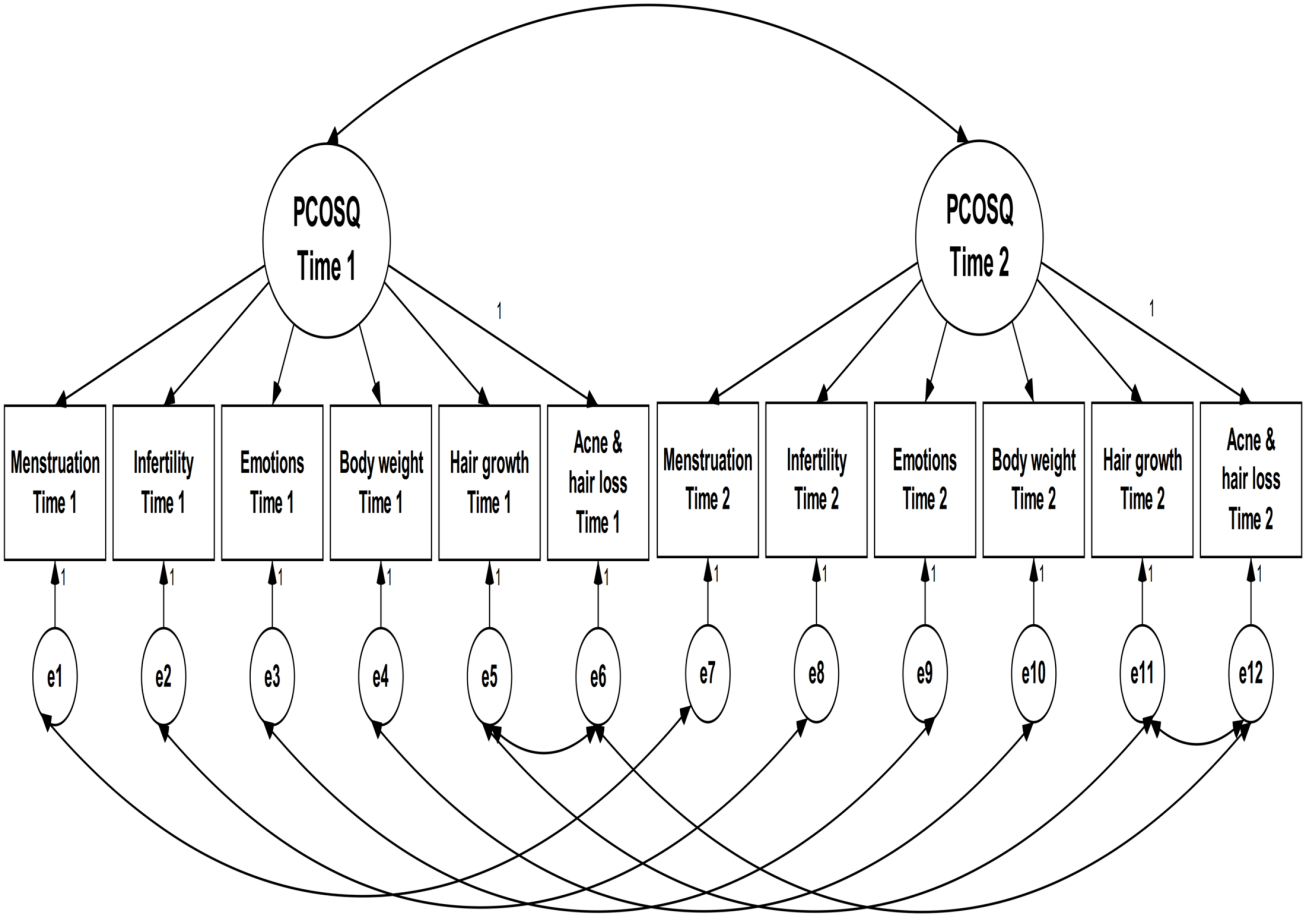
CFA Model 1 for Chi-PCOSQ.

Measurement invariance was examined using confirmatory factor analysis (CFA). Unlike exploratory factor analysis which is used to explore the optimal underlying structure of an instrument, CFA is used when a priori structure of an instrument is given [28]. That is, we used CFA to verify whether the priori structure is supported or not. Because the construct of Chi-PCOSQ has been investigated using EFA [15], we intended to use CFA to confirm the structure suggested by the previous study [15]. When using the CFA, we adopted the maximum likelihood estimator to gain the χ^2^ of anticipated (i.e., our priori structure for Chi-PCOSQ; Fig 1) and observed covariance matrices (i.e., correlating all the subscales of the Chi-PCOSQ); data-model fit can then be computed based on the difference between the two models (i.e., anticipated *versus* observed covariance matrices). Then, we used following steps to test the measurement invariance: (1) the data-model fit was examined in two baseline models (one at pre-test and the other at post-test), and the modification index (i.e., based on change of χ^2^ to revise the priori structure) was used if necessary; (2) based on the structure of the baseline model, the data-model fit of the configural model (all factor loadings and item intercepts were freely estimated; Fig 2: CFA Model 1 for Chi-PCOSQ) was examined across time; (3) the factor loadings of a given domain were constrained to be equal across time (i.e., the factor loading of Menstruation at pre-test was constrained to be the same as that at post-test, the factor loading of Infertility at pre-test was constrained to be identical to that at post-test, and so on; Model 2) and the data-model fit was examined; (4) the item intercepts of a given domain were constrained to be equal across time (i.e., the intercept of Menstruation at pre-test was constrained to be the same as that at post-test, the intercept of Infertility at pre-test was constrained to be identical to that at post-test, and so on; Model 3) and the data-model fit was examined; (5) Models 2 and 1 were compared to examine scalar invariance; (6) Models 3 and 2 were compared to examine metric invariance. Specifically, for the steps (5) and (6), we used the differences of data-model fit indices between the compared models (Models 2 *versus* 1; Models 3 *versus* 2). For all CFA models, we used each domain as the observed variable and one latent construct of the overall HRQoL because we tried to follow the principle of parsimony for CFA. Four indices were used for the data-model fit: a nonsignificant χ^2^ test, CFI > 0.9, a root-mean-square error of approximation (RMSEA) < 0.1, and a standardized root mean square residual (SRMR) < 0.08 [29, 30]. In addition, the following indices of measurement invariance were adopted: a nonsignificant Δχ^2^ test; ΔCFI > -0.01 with ΔRMSEA < 0.015 (for both scalar and metric invariances), and ΔSRMR < 0.03 (for scalar invariance) or < 0.01 (for metric invariance) [31, 32]. However, we did not attempt to use the criterion of nonsignificant χ^2^ and Δχ^2^ tests because they have the shortcoming of being too sensitive to a large sample size [31, 33]. Data description and responsiveness regarding paired *t*-tests of Chi-PCOSQ were analyzed using SPSS 17.0 for Windows (SPSS Inc., Chicago), responsiveness in terms of SRM was determined using G*Power 3.1.5 [34], and CFA was conducted using AMOS 7.0 (SPSS Inc., Chicago).

## Results

### Participant characteristics

A total of 102 patients who met study inclusion criteria were included, in which only 50 patients completed at least twice Chi-PCOSQ assessments during study period. So, there were 102 patients used for CFA and 50 patients for assessing the responsiveness and longitudinal validity of Chi-PCOSQ. The baseline mean ± SD age was 30.40 ± 5.57 years for the group used to assess responsiveness to change and longitudinal validity (n = 50) and 29.57 ± 5.50 years for the group used to assess CFA and measurement invariance (n = 102) (Table 1). More than three fourths of the participants had an educational level of college and above. Most participants were not currently smokers or drinkers, and around 70% of the participants had sexual experience (Table 1).

**Table 1:** Participants characteristics at baseline.

	For responsiveness and longitudinal validity (n = 50)	For confirmatory factor analysis (n = 102)
Age (year) (mean ± SD)	30.40 ± 5.57	29.57 ± 5.50
Educational level (≥ college)	38 (76.0%)	85 (83.3%)
Currently smoker (yes)	3 (6.0%)	4 (4.0%)
Currently drinker (yes)	8 (16.0%)	24 (23.5%)
Sexual experience (yes)	37 (74.0%)	69 (67.6%)

### Responsiveness to change

For the group used to assess responsiveness, the interval between the pre- and post-test dates was 3.60 ± 1.96 months. The participants showed clinical improvement after metformin treatment according to the 2-hour glucose and insulin data (pre-test 2-hour glucose = 120.22 ± 30.68 mg/dL, post-test 2-hour glucose = 106.20 ± 29.90 mg/dL, *t* (49) = 3.65, *p* = 0.001; pre-test 2-hour insulin = 74.94 ± 50.88 μIU/mL, post-test 2-hour insulin = 47.84 ± 29.19 μIU/mL, *t* (49) = 5.46, *p* < 0.001). With improved 2-hour glucose and insulin levels, we also found significantly increased Chi-PCOSQ scores in all domains (*t* (49) = 2.72 to 3.87; *p* < 0.01) except for hair growth (*t* (49) = 0.88; *p* = 0.39). The total score of Chi-PCOSQ also showed a significant improvement (*t* (49) = 5.20; *p* < 0.001). Moreover, the SRM values indicated that half of the domains (3 of 6) and the total score of Chi-PCOSQ had a medium responsiveness. In contrast, the responsiveness of WHOQOL-BREF was weak: three of the four domain scores were nonsignificant between pre- and post-test with trivial effects (Table 2).

**Table 2:** Responsiveness of Chi-PCOSQ and WHOQOL-BREF (n = 50).

Instrument (Domain)	Pretest score mean (SD)	Posttest score mean (SD)	*t* (*p*); *df* = 49	SRM
**Chi-PCOSQ**	**24.66 (5.94)**	**27.36 (6.36)**	**5.20 (< 0.001)**	**0.74**
(Menstruation)	3.67 (1.51)	4.41 (1.41)	3.63 (0.001)	0.51
(Infertility)	3.59 (1.99)	4.22 (2.00)	3.56 (0.001)	0.50
(Emotions)	4.15 (1.52)	4.60 (1.56)	3.78 (< 0.001)	0.53
(Body weight)	3.41 (1.57)	3.81 (1.48)	2.72 (0.009)	0.39
(Hair growth)	5.28 (1.66)	5.40 (1.70)	0.88 (0.39)	0.12
(Acne & hair loss)	4.54 (1.57)	4.94 (1.26)	3.41 (0.001)	0.48
**WHOQOL-BREF**	**55.29 (6.72)**	**56.15 (8.11)**	**1.81 (0.08)**	**0.26**
(Physical)	14.53 (1.88)	14.99 (2.09)	2.63 (0.011)	0.37
(Psychological)	12.67 (2.25)	12.88 (2.51)	1.28 (0.21)	0.18
(Social)	14.12 (1.90)	14.12 (2.07)	0.00 (1.00)	0.00
(Environment)	13.97 (1.83)	14.15 (2.23)	1.04 (0.30)	0.15

Abbreviations: SD: standard deviation, Chi-PCOSQ: Chinese version of Polycystic Ovary Syndrome Health-related Quality of Life Questionnaire, *df*: degree of freedom, SRM: standardized response mean, which was computed as the mean change scores divided by the standard deviation of the change.

Except for two domains (Infertility and Emotions), all the changes of domain scores and the total scores were negatively and significantly correlated with the change of 2-hour glucose. However, only the changes of the Acne & hair loss domain score and the total score were negatively and significantly correlated with the change of 2-hour insulin (Table 3).

**Table 3:** Longitudinal validity of Chi-PCOSQ (n = 50).

Change of Chi-PCOSQ score	Change of 2-hour glucose	Change of 2-hour insulin
	*r* (*p*)	*r* (*p*)
Chi-PCOSQ total score	−0.57 (< 0.001)*	−0.29 (0.045)*
Menstruation	−0.45 (0.001)*	−0.12 (0.40)
Infertility	−0.07 (0.64)	−0.05 (0.75)
Emotions	−0.26 (0.07)	−0.07 (0.65)
Body weight	−0.33 (0.02)*	−0.23 (0.10)
Hair growth	−0.40 (0.004)*	−0.23 (0.10)
Acne & hair loss	−0.54 (< 0.001)*	−0.36 (0.009)*

*p < 0.05

### Confirmatory factor analysis and measurement invariance

The modification index suggested correlating the uniqueness of the Hair growth domain with that of the Acne & hair loss domain in both baseline models (one for pre-test and the other for post-test). Therefore, we correlated the uniqueness in all CFA models. The results show that all fit indices of the two baseline models were acceptable, except for the slightly high RMSEA values (0.107 and 0.101). All fit indices of the models examining measurement invariance were satisfactory, except for the slightly high SRMR values (0.087 to 0.088). In addition, the model comparisons (nonsignificant Δχ^2^ tests, ΔCFI > −0.01, ΔRMSEA and ΔSRMR < 0.02) indicate that the measurement invariance of Chi-PCOSQ is supported across time (Table 4).

**Table 4:** Measurement invariance of Chi-PCOSQ across time.

**Model**	**χ**^**2**^ **(*df*)**	***p***	**CFI**	**RMSEA**	**SRMR**
Model at pre-test	17.17 (8)	0.03	0.930	0.107	0.064
Model at post-test	16.22 (8)	0.04	0.951	0.101	0.052
Model 1 (Configural)	77.62 (45)	0.002	0.970	0.085	0.088
Model 2 (Metric invariance)	81.20 (50)	0.003	0.971	0.079	0.088
Model 3 (Scalar invariance)	92.52 (56)	0.002	0.966	0.080	0.087
**Model comparisons**	**Δχ**^**2**^ **(Δ*df*)**	***p***	**ΔCFI**	**ΔRMSEA**	**ΔSRMR**
Models 2 and 1	3.58 (5)	0.61	−0.001	0.006	−0.0002
Models 3 and 2	11.31 (6)	0.08	0.005	−0.001	0.0004

Notes: The uniqueness of the Hair growth domain was correlated to that of the Acne & hair loss domain in all models.

Model 2 constrained all domain loadings to be invariant across pre- and post-test.

Model 3 constrained all domain intercepts to be invariant across pre- and post-test.

Abbreviations: CFI: comparative fit index, RMSEA: root-mean-square error of approximation, SRMR: standardized root mean square residual.

## Discussion

In a sample of ethnic Chinese women with PCOS, our results showed that Chi-PCOSQ was responsive to clinical improvement. However, WHOQOL-BREF, a generic HRQoL measurement, was not responsive. The longitudinal validity and the structure of Chi-PCOSQ were confirmed for a sample of Chinese women with PCOS. Also, Chi-PCOSQ demonstrated measurement invariance across time, implying its stability over time.

The present study showed that overall Chi-PCOSQ was sensitive to clinical changes (i.e., improved 2-hour glucose and insulin data) after initiation of metformin treatment. As for individual domains, the responsiveness was moderate for the Emotions, Infertility, and Menstruation domains, and trivial for the Hair growth and Body weight domains. The above results agree with the findings of Guyatt et al., who evaluated the responsiveness of the original PCOSQ and found treatment-related differences for the Emotions, Infertility, and Menstruation domains, but not for the Hair growth and Body weight domains [11]. Guyatt et al. offered two possible reasons to explain the inability of the original PCOSQ to detect clinical changes in hair growth and body weight. First, the items in PCOSQ regarding hair growth and body weight may be insensitive. Second, patients with PCOS did not have any changes in the clinical characteristics of hair growth and/or body weight before and after treatment [11]. We agree with these statements; however, these results were based on troglitazone, whereas metformin was used in the present study. Hence, future studies may want to examine whether metformin has any effects on hair growth, which has not been done yet.

In addition to the above two reasons, we propose another reason specifically for women of Chinese ethnicity: hirsutism and body weight problems are relatively low in Chinese patients with PCOS. Previous studies have shown that the prevalence of hirsutism in Chinese women with PCOS was in general low [35]; fewer than 10% of Chinese patients had a modified Ferriman-Gallwey (mF-G) score of > 5, which indicates clinical signs of excess androgen [36]. In fact, our study population reported few body hair problems and on average had a relatively high hair growth domain score for Chi-PCOSQ (i.e., implying less concern or psychological distress due to excess body hair) before and after treatment. Therefore, fewer excess body hair problems and a lack of clinical change in hair growth after metformin treatment in our study patients might explain the low sensitivity (responsiveness) of the hair growth domain for Chi-PCOSQ.

Previous studies have shown an effect of metformin on body weight [23, 24]. The body weight problem in our study patients was modest, with about 61% having decreased body weight after treatment. Although overweight or obesity is a common clinical presentation in PCOS women, Chinese women with PCOS are more likely to be slender as compared to Caucasian patients [21]. This implies that concern about body weight might be less problematic in Chinese women with PCOS. However, it might be possible that, although clinical change in body weight was sufficient to be measured in our study patients, it may not reflect the patients’ perspective about their body weight (e.g., patients may have high expectation of their body image). Nevertheless, the present study showed that Chi-PCOSQ was sensitive to clinical changes in PCOS patients, but that WHOQOL-BREF was not. This result confirms that a disease-specific instrument is more sensitive in detecting disease-specific clinical changes as compared to generic measurements. Therefore, Chi-PCOSQ might help clinicians identify changes of HRQoL associated with the symptoms of PCOS and its treatment.

The longitudinal validity analysis of Chi-PCOSQ showed that the changes of the objective indicators (2-hour glucose and insulin levels) were negatively correlated with the patients’ HRQoL. That is, our participants had higher HRQoL when their endocrine disorders were improved. 2-hour glucose and insulin levels have been used to screen PCOS patients because around 80% of PCOS women have abnormal 2-hour insulin levels, while almost all women without PCOS have normal 2-hour insulin levels [37]. Moreover, elevated levels of 2-hour glucose and insulin are indicators of impaired glucose tolerance and insulin resistance in PCOS patients and are thus used to predict patients’ risks for metabolic syndrome and diabetes [37]. As a result, 2-hour glucose and insulin levels are important clinical indicators for PCOS patients. Instead of using 2-hour glucose and insulin levels, Guyatt et al. evaluated the longitudinal validity of the original PCOSQ through hair growth (via an mF-G score), menstruation cyclicity, and free testosterone levels as indicators for clinical changes [11]. Their results showed weak correlations, but in the anticipated direction, between changes in PCOSQ score and changes in the clinical measurements [11]. Excess body hair growth, hirsutism, menstrual irregularity and hyperandrogenism may not manifest in all PCOS patients, which might explain the unimpressive longitudinal validity of PCOSQ reported by Guyatt et al [11]. In addition, the prevalence of hirsutism is low in Chinese women with PCOS, and the mean of the mF-G score in Chinese women with PCOS is 3–4 [38]. Fewer than half of Chinese patients with PCOS had clinical or biochemical hyperandrogenism [39], which appears to be age- and body mass index (BMI)-dependent in Taiwanese women with PCOS (i.e., increased BMI with elevated total testosterone levels) [40, 41]. Therefore, using 2-hour glucose and insulin data as indicators seems appropriate for assessing the longitudinal validity of Chi-PCOSQ in Chinese women with PCOS.

To the best of our knowledge, the measurement structure of the original PCOSQ has never been explored. The present study confirmed the adequate fit of the measurement structure of Chi-PCOSQ to Chinese women with PCOS. It incorporates three additional items, namely concerns about acne, hair loss, and getting diabetes, into the original PCOSQ. Although acne and hair loss are common in PCOS women, patients’ concerns and emotional disturbances associated with these clinical presentations were not addressed in the original PCOSQ. Also, increased risk for developing diabetes has been noticed in previous studies of Asian women with PCOS, which might lead to patients’ worries about getting diabetes. By adding the items of acne, hair loss, and fear of getting diabetes, our CFA results suggested that the measurement structure of Chi-PCOSQ fit Chinese culture. More importantly, our results of measurement invariance suggest that patients with PCOS interpret measurement items of Chi-PCOSQ the same across time, whether they receive treatment or not. Hence, clinicians should feel comfortable using Chi-PCOSQ for assessing the HRQoL of patients with PCOS at different time points. Chi-PCOSQ is expected to improve our understanding of the HRQoL of Chinese women with PCOS.

Several study limitations need to be addressed. Firstly, the study participants were from a single medical center in Taiwan, and thus may represent only a part of the Chinese population affected by PCOS. Secondly, the study participants were all treated with metformin and no placebo control group was included. Therefore, we cannot ascertain whether clinical improvement was from metformin efficacy or just changed over time. Thirdly, the HRQoL responses were self-reported by patients, and thus there might have been self-reporting bias. Lastly, although 2-hour glucose and insulin data can serve as objective indicators of the clinical changes associated with PCOS, other measures (e.g., physical examinations of acne and hirsutism, body weight, serum total testosterone, menstrual regularity) of the clinical changes of PCOS should be considered in future research.

## Conclusion

This study validated Chi-PCOSQ in terms of its responsiveness, longitudinal validity, and measurement invariance. Chi-PCOSQ is sufficiently sensitive in detecting clinical changes and the change of Chi-PCOSQ scores was correlated with the direction of clinical improvement. Our results also confirm that the measurement items of Chi-PCOSQ fit ethnic Chinese women with PCOS and that the patients’ interpretation of measurement items of Chi-PCOSQ is stable over time. Therefore, Chi-PCOSQ is a culturally appropriate HRQoL instrument for ethnic Chinese women affected by PCOS. Future research should be carried out to validate Chi-PCOSQ in Chinese women with PCOS across diverse geographic regions and social economic statuses. A cross-cultural comparison study of the HRQoL of PCOS patients could be conducted using Chi-PCOSQ to improve our understanding of the differences in psychological disturbances due to symptoms of PCOS across ethnicities.

## Supporting Information

S1 Appendix Chinese Version of Polycystic Ovary Syndrome Health-related Quality of Life Questionnaire (Chi-PCOSQ). (DOCX)
